# Fundamental Studies of Novel Zwitterionic Hybrid Membranes: Kinetic Model and Mechanism Insights into Strontium Removal

**DOI:** 10.1155/2014/485820

**Published:** 2014-10-27

**Authors:** Wen Zhu, Junsheng Liu, Meng Li

**Affiliations:** Key Laboratory of Membrane Materials & Processes, Department of Chemical and Materials Engineering, Hefei University, 99 Jinxiu Avenue, Hefei Economic and Technological Development Zone, Hefei 230601, China

## Abstract

A series of zwitterionic hybrid membranes were prepared via the ring opening of 1,3-propanesultone with the amine groups in the chains of TMSPEDA and a subsequent sol-gel process. Their kinetic models for strontium removal were investigated using three two-parameter kinetic equations (i.e., Lagergren pseudo-first order, pseudo-second order, and Elovich models). Adsorption mechanism was evaluated using intraparticle diffusion model, diffusion-chemisorption model, and Boyd equation. It was found that the adsorption of strontium ions on these zwitterionic hybrid membranes fitted well with the Lagergren pseudo-second order model. Mechanism insights suggested that diffusion-chemisorption was one of the main adsorption mechanisms. Boyd equation exhibited that film-diffusion mechanism might be the control process during the starting period. These findings are very useful in strontium removal from the stimulated radioactive wastewater.

## 1. Introduction

As one of the most promising energy supply approaches for fossil fuels, nuclear power technology has caught escalating interests. As a result, nuclear power station gained rapid growth throughout the world. Meanwhile, the amount of spent radioactive wastewater was also increased to a new level. This radioactive wastewater contains various radionuclides, such as strontium (^90^Sr), cobalt (^60^Co), and cesium (^137^Cs), which will threat the health of human body and various organisms in the world [1–3]. The nuclear accident especially at the Fukushima Daiichi Nuclear Power Plant (Japan) has caused great panic among the inhabitants in Asia-pacific region and worried the people around the world [4, 5]. Thus the effective treatment of radionuclides from the spent radioactive wastewater challenges the researchers and engineers. Among the radionuclides, water pollution caused by strontium-90 has captured great attention. This is because strontium (^90^Sr) has similar properties to calcium and can induce cancer and various diseases [1, 2]. The removal of strontium ions from water is thus significantly important.

To delete or restrain the potential danger caused by strontium ions, various innovative strategies are recently designed to remove strontium ions from water [1, 2, 6–8]. Among these methods, membrane adsorption exhibited obvious advantages over the others [5, 8]. However, the theoretical fundamentals of membrane adsorption for strontium removal were conducted insufficiently. Particularly, the mechanism of membrane adsorption cannot be deeply understood. Its application is thus blocked. Further study on membrane adsorption for strontium removal is therefore necessary.

Recently, much effort was made to fabricate new hybrid membranes as adsorbents to remove metals from water [9–11]. These hybrid membranes exhibited excellent adsorption properties for heavy-metal ions in aqueous solution. Particularly, as one important type of polymeric materials or membranes, zwitterionic ones simultaneously contain both cations and anions (or cation-exchange and anion-exchange groups); these ionic groups are arranged as the pendent-side chain structures. Such unique molecular structures provide them with excellent property performances. As the negatively charge membranes can be used to remove cations from aqueous solution, zwitterionic ones expect to be applied to remove metal ions from water by adsorptive separation. Therefore, zwitterionic polymers and their derivatives attracted much interest and have been successfully applied to remove metal cations from water, indicating promising application prospects in separation field [12, 13]. For example, Wang et al. [12] used a silica gel functionalized with a ditopic zwitterionic Schiff base ligand to adsorb Cu(II) and SO_4_^2−^ ions simultaneously. Our interest in zwitterionic hybrid membranes and the effective disposal of heavy metals from water motivates us to do more work.

Therefore, to extend the application field of membranes and develop a new type of zwitterionic hybrid membranes for the removal of strontium (Sr^2+^) ions from the stimulated radioactive wastewater, herein, a series of novel zwitterionic hybrid membranes were prepared via the ring opening of 1,3-propanesultone (PS) with the amine groups in the chains of TMSPEDA and a subsequent sol-gel process. Their kinetic models and adsorption mechanism for strontium removal were examined. The novelty of this investigation is that (1) the anionic and cationic groups were grafted on the molecular chains the ring-opening reaction of 1,3-propanesultone (PS) with the amine groups in the chains of TMSPEDA; (2) the application of zwitterionic hybrid membranes was extended to the new field of radionuclide removal, and (3) as typical examples, the kinetic models and adsorption mechanism of strontium ions were investigated for the potential removal of radionuclides from the stimulated radioactive wastewater.

## 2. Experimental 

### 2.1. Materials

*N*-[3-(Trimethoxysilyl)propyl] ethylenediamine (TMSPEDA, purity ≥ 95%) was purchased from Jiangsu Chenguang Coincident Dose Co., Ltd. (Danyang City, China), and used without further purification. Poly(vinyl alcohol) (PVA) was purchased from Shanghai Chemical Reagent Co., Ltd. (Shanghai City, China), and used as received. 1,3-Propanesultone (PS, purity ≥ 99%), SrCl_2_, and other reagents were of analytical grade and used as received.

### 2.2. Membrane Preparation

The preparing procedure for zwitterionic hybrid membranes can be described in detail as follows. First, 50 g PVA was dissolved in deionized water and stirred vigorously for 5 h at 92°C to produce the 5% aqueous PVA solution. Second, prescribed amount of TMSPEDA reacted with 1,3-propanesultone (the weight ratio of TMSPEDA : PS in samples A–D was 3.0 : 0, 3.0 : 1.5, 3.0 : 3.0, and 3.0 : 6.0, resp.) in a DMF solution at room temperature and was stirred for 5 h to prepare the hybrid precursor of sol-gel process. Third, the above-prepared hybrid precursor was added dropwise into the 5% PVA aqueous solution and stirred for additional 5 h. Subsequently, it was aged for additional 5 h to obtain the coating solution. The coating solution for membrane preparation was thus obtained.

To obtain the zwitterionic hybrid membranes, the previously prepared coating solution was coated on a glass plate and air dried at room temperature for additional 5 days. The zwitterionic hybrid membrane for strontium removal could thus be achieved.

### 2.3. Adsorption Experiment

The adsorption capacity (*q*_*t*_) of strontium ions on the previously prepared zwitterionic hybrid membranes at contact time (*t*) can be calculated using the following:

(1) $$\begin{array}{c} q_{t}=\frac{\left(C_{0}-C_{R}\right)V}{W}, \end{array}$$

where *V* is the volume of aqueous SrCl_2_ solution (mL), *C*_0_ and *C*_*R*_ are the concentration of initial and remaining SrCl_2_ (mol/L), respectively, and *W* is the weight of samples (g).

## 3. Results and Discussion 

### 3.1. Membrane Preparation

**Scheme 1:**
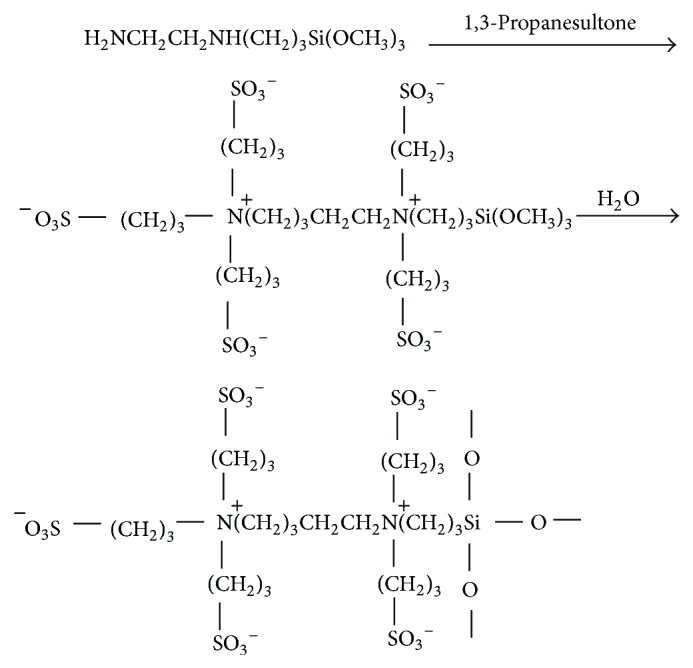
The production of hybrid precursor (nonstoichiometric balance).

The hybrid precursor for preparing the zwitterionic hybrid membranes was obtained via sol-gel process, in which the ring-opening reaction of PS with the amine groups in the chains of TMSPEDA was performed. Accordingly, the –N^+^– and –SO_3_^−^ groups were produced in the molecular chains of hybrid precursor (Scheme 1). Since such type of hybrid precursor simultaneously contains anionic and cationic groups, the hybrid membranes prepared from these hybrid precursors can be defined as zwitterionic ones.

### 3.2. Adsorption Capacity versus Contact Time

To simplify the adsorption process, only the adsorption of strontium ions was considered in this case. We hope that some preliminary adsorption data and typical models can be achieved via the measurement of the single strontium ions.

**Figure 1:**
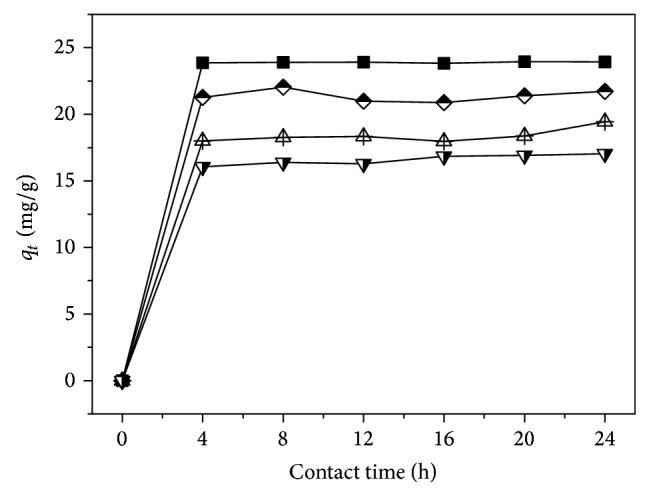
The plot of adsorption capacity of strontium ions on samples A (solid square), B (half-filled diamond), C (center up triangle), and D (half-filled down triangle) versus contact time; the concentration of aqueous SrCl_2_ solution was 0.004 mol/L at 65°C and pH 10.

Figure 1 presents the relationship of adsorption capacity (*q*_*t*_) versus contact time (*t*).

As shown in Figure 1, it is clear that, for samples A–D, the *q*_*t*_ values all increased with the elapsed contact time and reached an equilibrium state as the contact time exceeded 4 h. From such change trends, it can be deduced that the saturation adsorption time was around 4 h. Meanwhile, for the individual sample, it can be seen that the *q*_*t*_ value decreased from sample A to D, suggesting that the grafting of –SO_3_^−^ groups on the molecular chains does not favor the increase in the adsorption capacity of strontium ions. The reason can be ascribed to the strong dissociation of –SO_3_^−^ groups and the electrostatic repulsion from the –N^+^– groups on strontium ions. In addition, the difference of complex existing in the –SO_3_^−^ groups and –NH_2_ groups with the metal ions might also be responsible for such trend.

To obtain the optimal fitting kinetic model for strontium removal, as typical examples, these adsorption data were modeled using typical two-parameter kinetic equations, such as Lagergren pseudo-first order and pseudo-second order kinetic models, and Elovich equations, in which the adjusted linear regression (*R*_adj_^2^) was used to evaluate which model is fitting better or worse. Moreover, to have insight into the adsorption mechanism of strontium ions on samples A–D, these experimental data were analyzed using intraparticle diffusion model, diffusion-chemisorption model, and Boyd equation.

### 3.3. Lagergren Kinetic Model

Presently, it is well accepted that Lagergren kinetic equation is a helpful tool to evaluate the adsorption performances of an adsorbent. Typically, Lagergren pseudo-first order and pseudo-second order kinetic models can be linearly expressed as (2b) and (2d), respectively [14–16]:

(2a) $$\begin{array}{c} q_{t}=q_{e}\left(1-e^{-k_{1}t}\right) \end{array}$$

or

(2b) $$\begin{array}{c} \ln\left(q_{e}-q_{t}\right)=\ln q_{e}-k_{1}t, \end{array}$$

(2c) $$\begin{array}{c} q_{t}=\frac{q_{e}^{2}k_{2}t}{\left(1+q_{e}k_{2}t\right)} \end{array}$$

or

(2d) $$\begin{array}{c} \frac{t}{q_{t}}=\frac{1}{k_{2}q_{e}^{2}}+\frac{t}{q_{e}}, \end{array}$$

where *k*_1_ (h^−1^) and *k*_2_ (g mg^−1^ h^−1^) are the rate constant of pseudo-first order and pseudo-second order kinetic models, respectively, and *q*_*t*_ and *q*_*e*_ (mg/g) are the adsorption capacity of metal ions at time *t* (h) and at equilibrium state, respectively.

**Figure 2:**
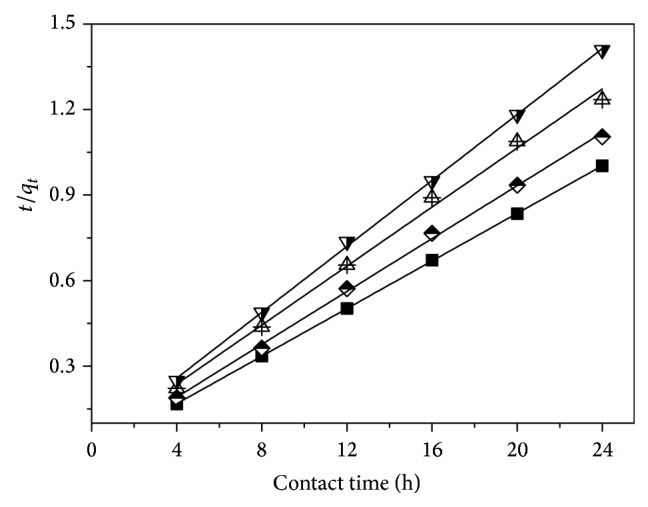
Lagergren pseudo-second order kinetic model for the adsorption of strontium ions on samples A (solid square), B (half-filled diamond), C (center up triangle), and D (half-filled down triangle).

Figure 2 illustrates the Lagergren pseudo-second order kinetic model for the adsorption of strontium ions on samples A–D. The related model parameters are summarized in Table 1.

As listed in Table 1, it is clear that the experimental data fit well with the Lagergren pseudo-second order model (*R*_adj_^2^ > 0.99). In contrast, they fit worse with the pseudo-first one (*R*_adj_^2^ < 0.55; the plot of ln⁡(*q*_*e*_ − *q*_*t*_) versus *t* is not presented in the text). Meanwhile, it can be observed that the *q*_*e*,cal_ values from the Lagergren pseudo-second order kinetic model were very close to the *q*_*e*,exp⁡_. These findings suggest that Lagergren pseudo-second order kinetic model can be used to describe the adsorption performances of strontium ions on the prepared zwitterionic hybrid membranes.

### 3.4. Elovich Model

Elovich equation [17, 18] usually was used to study the kinetics of chemisorption of gases on the solid surface. Presently, it was reported [17] that such kinetic equation can also be used to investigate the liquid-state sorption of an adsorbent and expressed linearly as

(3) $$\begin{array}{c} q_{t}=a+b\ln\left(t\right), \end{array}$$

where *a* (mg/g) and *b* are the Elovich parameters, which can be obtained from the intercept and slope of straight line.

**Figure 3:**
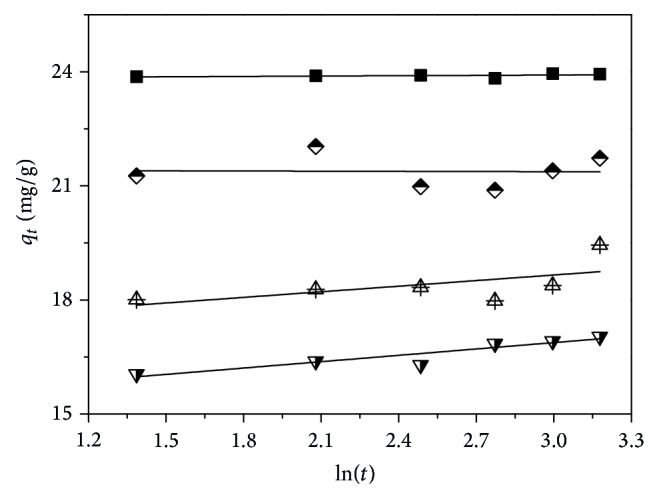
Elovich model for the adsorption of strontium ions on samples A (solid square), B (half-filled diamond), C (center up triangle), and D (half-filled down triangle).

The Elovich model for the adsorption of strontium ions on samples A–D was carried out and is given in Figure 3. From Figure 3, it can be noted that the linear fit curves did not exhibit better adaptability with the Elovich model (*R*_adj_^2^ < 0.80; these data were not listed in the text), demonstrating that the adsorption of strontium ions on samples A–D cannot be described using the Elovich model. This outcome reveals that chemical adsorption between the active sites on membrane surface and strontium ions did not exist. Such finding further evidences that the electrostatic effect between the ionic groups and strontium ions was the dominating factor, which will impact the adsorption behaviors of strontium ions.

### 3.5. Mechanism Insights

To explain the adsorption behaviors of metal ions on a solid-state adsorbent, it is important to gain insight into the adsorption mechanism. Presently, it is reported that intraparticle diffusion model [19, 20], diffusion-chemisorption model [21], and Boyd equation [22] are useful models to study the adsorption mechanism. To gain the adsorption mechanism of strontium ions on the prepared zwitterionic hybrid membranes, the experimental data were analyzed using these typical models.

#### 3.5.1. Intraparticle Diffusion

Intraparticle diffusion model [19, 20] usually was used to describe the transport property of metal ions from the solution to the interface, which can be expressed as

(4) $$\begin{array}{c} q_{t}=x_{i}+k_{p}t^{0.5}, \end{array}$$

where *q*_*t*_ (mg/g) is the adsorbed amount at time *t* (h), *k*_*p*_ (mg g^−1^ h^−1/2^) is the intraparticle diffusion rate constant, and *x*_*i*_ (mg/g) is the intercept of straight line, which is related to the boundary layer thickness.

It was proposed [19, 20] that if the plot of *q*_*t*_ versus *t* ^0.5^ gives a straight line, the adsorption process is solely controlled by intraparticle diffusion. In contrast, if the linear fitting exhibits multilinear curves, two or more steps will influence the adsorption process.

**Figure 4:**
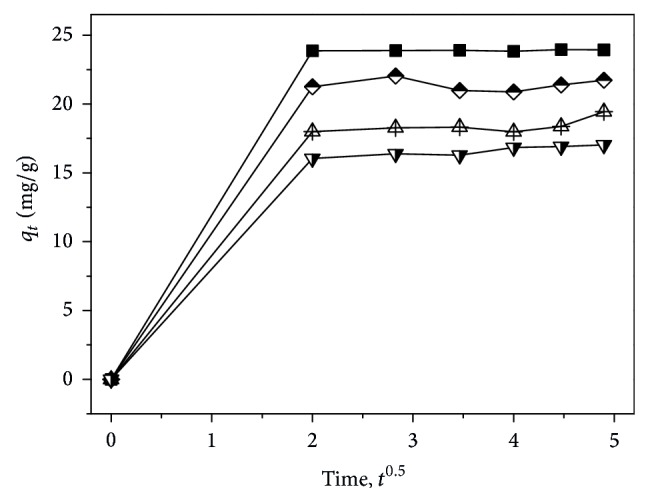
Intraparticle diffusion model for the adsorption of strontium ions on samples A (solid square), B (half-filled diamond), C (center up triangle), and D (half-filled down triangle).

The intraparticle diffusion model for the adsorption of strontium ions on samples A–D is exhibited in Figure 4.

As presented in Figure 4, it can be found that the curves did not exhibit a straight line. Two straight lines (i.e., in the range of 0–4 and 4–24 h) were clearly detected, suggesting that the adsorption of strontium ions on samples A–D was not governed uniquely by intraparticle diffusion; more adsorption processes possibly occurred. The starting stage can be assigned to the surface adsorption from the electrostatic attraction effect of –SO_3_^−^ groups in the zwitterionic hybrid membranes and strontium (Sr^2+^) ions in the aqueous solution. Since the effect of electrostatic attraction between the –SO_3_^−^ groups and Sr^2+^ ions dominated the starting period, such adsorption was faster. Meanwhile, the straight lines went through the origin (intercept *x*_*i*_ = 0); it can be concluded that the adsorption process during the starting period was solely controlled by intraparticle diffusion; that is, intraparticle diffusion is the rate-limiting step for the first adsorption process. From the slope of straight lines, it can be seen that the intraparticle diffusion rate constant *K*_*p*_ values of samples A–D were 11.9364, 10.63045, 9.0015, and 8.02445 mg g^−1^ h^−1/2^, respectively, indicating a downward trend from samples A to D.

Moreover, taking the second step into consideration, it can be observed that the straight lines did not go through the origin. Such trends imply that the effect of boundary layer thickness on the adsorption of strontium ions cannot be neglected. More influencing factors, such as mass transfer, will impact the adsorption process as discussed later.

#### 3.5.2. Diffusion-Chemisorption Model

It was proposed [22] that diffusion-chemisorption model can be used to describe the sorption of heavy metal ions onto the heterogeneous media, which can be expressed linearly as

(5) $$\begin{array}{c} \frac{t^{0.5}}{q_{t}}=\frac{1}{K_{\text{DC}}}+\frac{1}{q_{e}}t^{0.5}, \end{array}$$

where *K*_DC_ is the diffusion-chemisorption constant.

**Figure 5:**
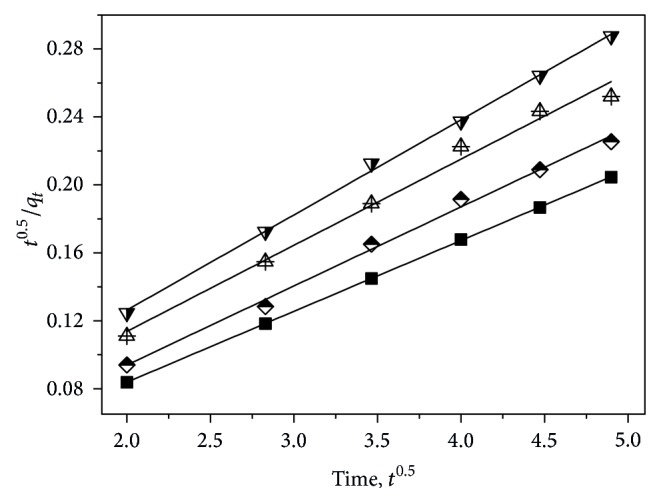
Diffusion-chemisorption model for the adsorption of strontium ions on samples A (solid square), B (half-filled diamond), C (center up triangle), and D (half-filled down triangle).

The diffusion-chemisorption model for the adsorption of strontium ions on samples A–D is exhibited in Figure 5. The related kinetic model parameters are tabulated in Table 2.

As shown in Figure 5, it can be noted that these curves fit well with the diffusion-chemisorption model (*R*_adj_^2^ > 0.98, cf. Table 2), suggesting that the adsorption of strontium ions on samples A–D can be described using the diffusion-chemisorption model; that is, the adsorption of strontium ions on samples A–D suffers from the heterogeneous environments of the prepared zwitterionic hybrid membranes. Moreover, comparing the *q*_*e*,cal_ values obtained by diffusion-chemisorption model with those obtained via Lagergren pseudo-second order kinetic model, it can be discovered that they had very close results although these calculated data were all larger than the *q*_*e*,exp⁡_ values. This finding demonstrates that it is reasonable using diffusion-chemisorption model to explain the adsorption of strontium ions on samples A–D.

#### 3.5.3. Boyd Equation

It was reported [21–24] that Boyd equation can be applied to determine the rate-controlling step for an adsorbent and expressed as

(6) $$\begin{array}{c} F=1-\left(\frac{6}{\pi^{2}}\right)\exp\left(-Bt\right), \end{array}$$

where *Bt* is the function of *F* and *F* is the fraction of solute adsorbed at different times, *t*. The *F* value can be calculated using

(7) $$\begin{array}{c} F=\frac{q_{t}}{q_{e}}, \end{array}$$

where *q*_*t*_ and *q*_*e*_ are the amount of adsorbed on the adsorbent at any time *t* and at equilibrium state, respectively.

The *Bt* values at different contact times, *t*, can be calculated using the following in the case of *F* > 0.85 [22–24]:

(8) $$\begin{array}{c} Bt=-0.4977-\ln\left(1-F\right). \end{array}$$

On the basis of *Bt* against contact time, *t*, the plot of Boyd model can be obtained. It was proposed [22–24] that if the plot of *Bt* versus *t* is a straight line and passes through the origin, it is the pore diffusion that controls the rate of mass transfer (or particle diffusion mechanism). In contrast, if the plot is nonlinear or linear but does not pass through the origin, film-diffusion or external mass transport will be the major dominating factor.

**Figure 6:**
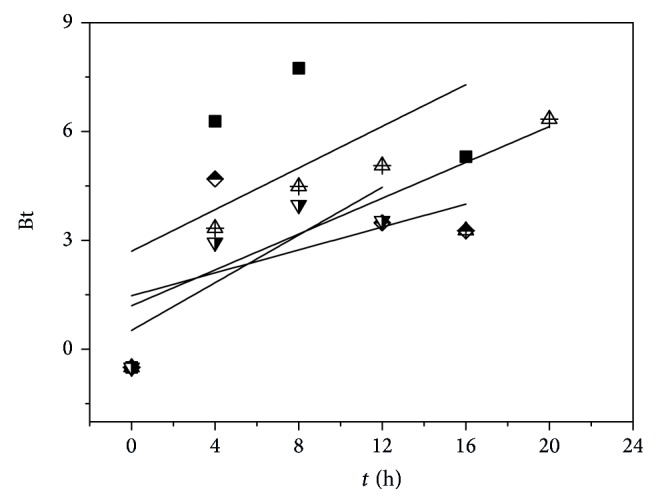
The plot of Boyd model for the adsorption of strontium ions on samples A (solid square), B (half-filled diamond), C (center up triangle), and D (half-filled down triangle).

Figure 6 presents the plot of Boyd model for the adsorption of strontium ions on samples A–D.

As shown in Figure 6, it can be noted that the curves are all nonlinear and do not pass through the origin. Based on these findings, it can be concluded that the adsorption of strontium ions on samples A–D is a film-diffusion mechanism.

## 4. Conclusions

The zwitterionic hybrid membranes were used as adsorbents to remove single strontium ions from the stimulated radioactive wastewater. The experimental data were analyzed using various typical models. The following results can be achieved.

1. It was discovered that the adsorption of strontium ions on the prepared zwitterionic hybrid membranes followed the Lagergren pseudo-second order model.
2. Elovich model revealed that chemisorption cannot be used to describe the adsorption behaviors of strontium ions on these zwitterionic hybrid membranes.
3. Mechanism studies demonstrated that intraparticle diffusion was not the solely controllable process, and diffusion-chemisorption might be more rational to explain the adsorption of strontium ions on these zwitterionic hybrid membranes. Meanwhile, Boyd equation showed that the adsorption was film-diffusion mechanism.

It should be emphasized that this paper mainly focuses on the adsorption of single Sr^2+^ ions using zwitterionic hybrid membranes as adsorbents, and little work is done to examine the adsorption of different metal ions in the competing mixed system. However, this does not mean that the adsorption of mixed cations is less important. In fact, for the practical applications of zwitterionic hybrid membranes in industrial processes, further research is needed to optimize the adsorption process so as to improve the selectivity of hybrid membranes for different metal cations in the competing mixed cations system. We believe that a satisfactory result will be obtained via the optimization of hybrid membrane natures, which will be our future job.

## Figures and Tables

**Table 1:** Lagergren pseudo-second order kinetic model parameters for strontium removal.

Sample	*k*_2_ (g mg^−1^ h^−1^)	*h* ^*^ (mg g^−1^ h^−1^)	*q*_*e*,cal_ (mg g^−1^)	*q*_*e*,exp_ (mg g^−1^)	*R*_adj_^2^
A	1.657	950.897	23.953	23.90	0.999
B	0.386	179.569	21.563	21.37	0.998
C	0.0898	33.527	19.318	18.40	0.994
D	0.126	37.843	17.296	16.58	0.999

∗Initial adsorption rate (*h*) = *k*_2_ *q*_*e*_^2^.

**Table 2:** Diffusion-chemisorption kinetic parameters for strontium removal.

Sample	Diffusion-chemisorption kinetic model			
	*K*_DC_ (mg g^−1^ h^−0.5^)	*q*_*e*,cal_ (mg g^−1^)	*q*_*e*,exp_ (mg g^−1^)	*R*_adj_^2^
A	2218.524	23.976	23.90	0.999
B	1153.257	21.482	21.37	0.994
C	81.156	19.717	18.40	0.986
D	70.181	17.848	16.58	0.998
